# Poly[(*μ*_6_-benzene-1,2,4,5-tetra­carboxyl­ato)bis­(1,10-phenanthroline-*κ* ^2^ *N*,*N*′)dimanganese(II)]

**DOI:** 10.1107/S1600536808016723

**Published:** 2008-06-13

**Authors:** Xin-Dong Jiang, Xiu-Bing Li, Bai-Wang Sun

**Affiliations:** a Ordered Matter Science Research Center, College of Chemistry and Chemical Engineering, Southeast University, Nanjing 210096, People’s Republic of China; b Department of Chemistry, Key Laboratory of Medicinal Chemistry for Natural Resources, Ministry of Education, Yunnan University, Kunming 650091, People’s Republic of China

## Abstract

The title polymeric compound, [Mn_2_(C_10_H_2_O_8_)(C_12_H_8_N_2_)_2_]_*n*_, was obtained by the reaction of manganese(II) chloride tetra­hydrate with benzene-1,2,4,5-tetra­carboxylic acid (H_4_bta) in aqueous solution. Each Mn^2+^ ion is coordinated in a distorted octa­hedral geometry by two N atoms from one 1,10-phenanthroline ligand and four O atoms [Mn—O = 2.116 (2)–2.237 (2) Å] from three bta^4−^ ligands, which also act as bridging groups between the Mn^2+^ ions.

## Related literature

For general background, see: Rao *et al.* (2000 ▶). For related structures, see: Aghabozorg *et al.* (2007 ▶); Chu *et al.* (2001 ▶); Liu & Ding (2007 ▶); Wu *et al.*, (2006 ▶).
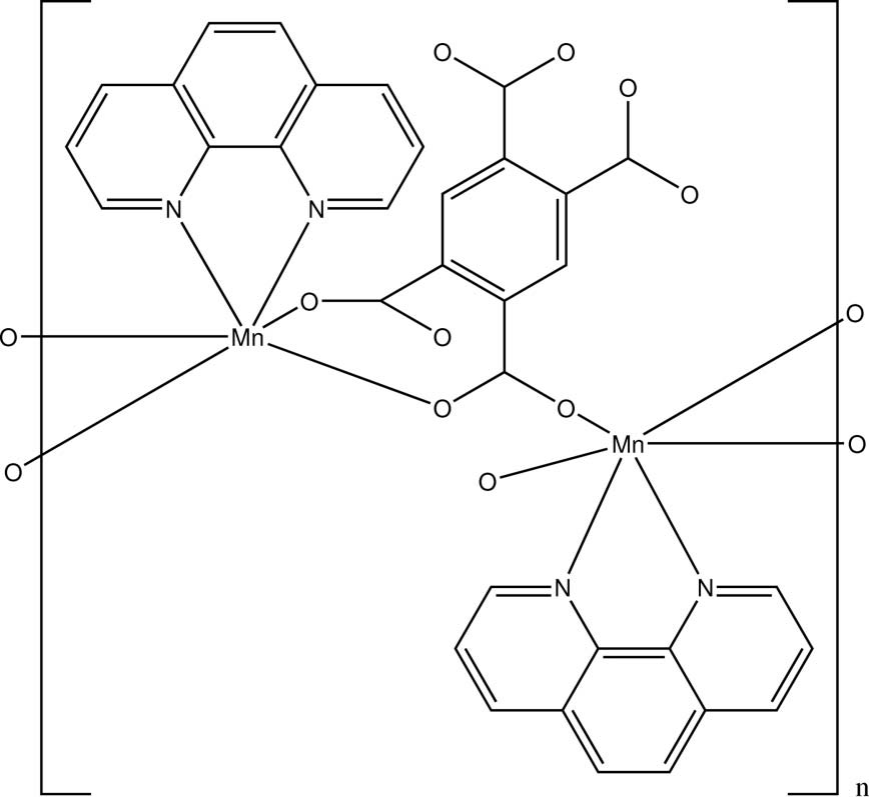


## Experimental

### 

#### Crystal data

[Mn_2_(C_10_H_2_O_8_)(C_12_H_8_N_2_)_2_]
*M* *_r_* = 360.20
Monoclinic, 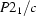

*a* = 7.5115 (7) Å
*b* = 19.8111 (19) Å
*c* = 9.6327 (9) Å
β = 112.027 (2)°
*V* = 1328.8 (2) Å^3^
*Z* = 4
Mo *K*α radiation
μ = 1.02 mm^−1^
*T* = 293 (2) K
0.22 × 0.20 × 0.18 mm

#### Data collection

Rigaku Scxmini CCD area-detector diffractometer
Absorption correction: multi-scan (*CrystalClear*; Rigaku, 2005 ▶) *T*_min_ = 0.796, *T*_max_ = 0.833
8026 measured reflections
2336 independent reflections
1853 reflections with *I* > 2σ(*I*)
*R*_int_ = 0.057

#### Refinement

*R*[*F* ^2^ > 2σ(*F* ^2^)] = 0.043
*wR*(*F* ^2^) = 0.107
*S* = 0.99
2336 reflections
217 parameters
H-atom parameters constrained
Δρ_max_ = 0.58 e Å^−3^
Δρ_min_ = −0.35 e Å^−3^

### 

Data collection: *CrystalClear* (Rigaku, 2005 ▶); cell refinement: *CrystalClear*; data reduction: *CrystalClear*; program(s) used to solve structure: *SHELXS97* (Sheldrick, 2008 ▶); program(s) used to refine structure: *SHELXL97* (Sheldrick, 2008 ▶); molecular graphics: *SHELXTL* (Sheldrick, 2008 ▶); software used to prepare material for publication: *SHELXTL*.

## Supplementary Material

Crystal structure: contains datablocks I, New_Global_Publ_Block. DOI: 10.1107/S1600536808016723/rt2017sup1.cif

Structure factors: contains datablocks I. DOI: 10.1107/S1600536808016723/rt2017Isup2.hkl

Additional supplementary materials:  crystallographic information; 3D view; checkCIF report

## Figures and Tables

**Table:** 

Mn1—O2	2.116 (2)
Mn1—O3	2.125 (2)
Mn1—O4	2.204 (2)
Mn1—O1	2.237 (2)
Mn1—N2	2.252 (3)
Mn1—N1	2.305 (3)

**Table:** 

O2—Mn1—O3	107.56 (9)
O2—Mn1—O4	81.59 (9)
O3—Mn1—O4	99.15 (8)
O2—Mn1—O1	96.86 (9)
O3—Mn1—O1	80.40 (8)
O4—Mn1—O1	178.19 (9)
O2—Mn1—N2	86.52 (9)
O3—Mn1—N2	156.89 (9)
O4—Mn1—N2	101.02 (9)
O1—Mn1—N2	79.78 (9)
O3—Mn1—N1	98.36 (9)
O4—Mn1—N1	85.10 (9)
O1—Mn1—N1	96.70 (9)
N2—Mn1—N1	72.38 (9)

Symmetry codes: (i) 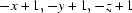; (ii) 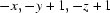.

**Fig. 1.**
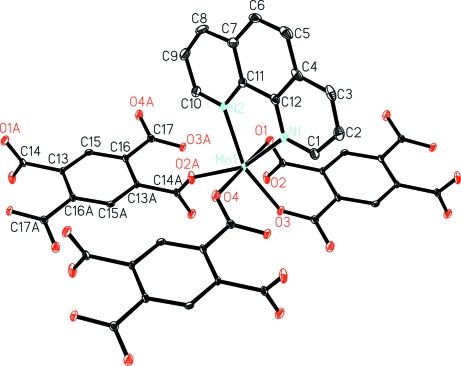
The molecular structure of the title molecule, with the atom-numbering scheme. Displacement ellipsoids are drawn at the 30% probability level.

**Fig. 2.**
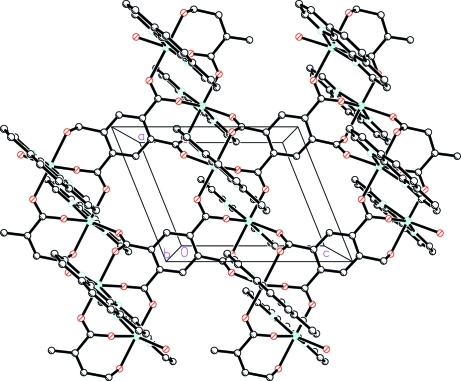
A view of the polymeric layer for compound (I).

**Fig. 3.**
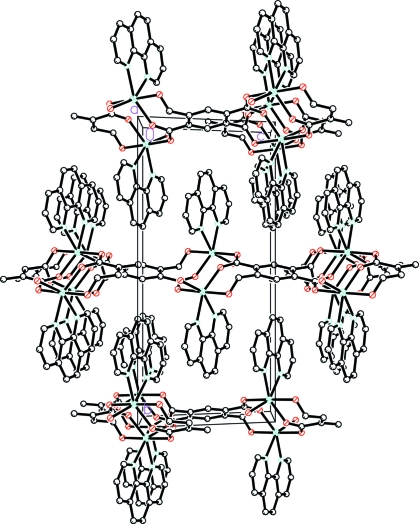
Crystal packing of the compound (I).

## supplementary crystallographic information

### Comment

The recent interest in the crystal engineering of special geometrical and topological coordination polymers arises from their potential application in catalysis, chemical absorption, magnetism and electrical conductivity (For related structures, see: Rao *et al.*, 2000). The benzene-1,2,4,5-teracarboxylate ligand (bta) as a multi-connecting ligand is also an excellent candidate for the structuring of coordination polymers, and comparatively few examples have been reported to date in relation to applying it to the building of coordination polymers (For details of the preparation of related compounds, see: Aghabozorg *et al.*, 2007; Chu *et al.*, 2001; Liu & Ding, 2007; Wu *et al.*, 2006). We report here the synthesis and crystal structure of the title complex, (I) (Fig. 1).

As shown in Fig.1, only one bta ligand is located in the crystallographic asymmetric unit, while each bta ligand is shared between three manganese(II) centres. The manganese atom is situated on an inversion centre and is coordinated in a *trans* mode by one chelated phen ligand [Mn—N = 2.252 (3) and 2.305 (3) Å] and four carboxylate oxygen atoms [Mn—O = 2.116 (2), 2.125 (2), 2.204 (2) and 2.237 (2) Å] (Table 1) from three distinct bta ligands. The coordination geometry around the Mn^II^ ion is slightly distorted octahedral. The O1 and O4 atoms occupy *trans* positions. Each bta ligand bridges to six manganese atoms to generate a two-dimensional sheet architecture, in which the carboxylate groups of the bta ligand all display a bridging mode (Fig. 2). Along the crystallographic *a*-axis, the manganese atoms are maintained in a pseudo-chain arrangement with a Mn···Mn distance of 4.611 Å (Fig. 3).

### Experimental

All reagents and solvents were used as obtained without further purification. MnCl_2_.4H_2_O (59 mg,0.3 mmol), H_4_bta (76 mg, 0.3 mmol) and NaOH (24 mg, 0.6 mmol) were dissolved in 10 ml of distilled water. The mixture was sealed in a Teflon-lined stainless steel vessel and kept at 443 K for one week. The vessel was gradually cooled to room temperature, and brown crystals suitable for crystallographic analysis were obtained after two weeks. These latter crystals were filtered, washed with water, and dried in air. Yield: 32 mg (30%) based on Mn.

### Refinement

Positional parameters of all H atoms were calculated geometrically and were allowed to ride on their corresponding parent C atoms, with *U*_iso_(H) = 1.2*U*_eq_(C).

### Crystal data

**Table:**

[Mn_2_(C_10_H_2_O_8_)(C_12_H_8_N_2_)_2_]	*F*_000_ = 728
*M**_r_* = 360.20	*D*_x_ = 1.800 Mg m^−^^3^
Monoclinic, *P*2_1_/*c*	Mo *K*α radiation λ = 0.71073 Å
Hall symbol: -P 2ybc	Cell parameters from 4526 reflections
*a* = 7.5115 (7) Å	θ = 3.3–26.0º
*b* = 19.8111 (19) Å	µ = 1.02 mm^−^^1^
*c* = 9.6327 (9) Å	*T* = 293 (2) K
β = 112.027 (2)º	Block, brown
*V* = 1328.8 (2) Å^3^	0.22 × 0.20 × 0.18 mm
*Z* = 4	

### Data collection

**Table:**

Rigaku Scxmini 1K CCD area-detector diffractometer	2336 independent reflections
Radiation source: fine-focus sealed tube	1853 reflections with *I* > 2σ(*I*)
Monochromator: graphite	*R*_int_ = 0.057
Detector resolution: 8.192 pixels mm^-1^	θ_max_ = 25.0º
*T* = 293(2) K	θ_min_ = 2.1º
thin–slice ω scans	*h* = −8→8
Absorption correction: multi-scan(CrystalClear; Rigaku, 2005)	*k* = −23→23
*T*_min_ = 0.796, *T*_max_ = 0.833	*l* = −11→10
8026 measured reflections	

### Refinement

**Table:**

Refinement on *F*^2^	Secondary atom site location: difference Fourier map
Least-squares matrix: full	Hydrogen site location: inferred from neighbouring sites
*R*[*F*^2^ > 2σ(*F*^2^)] = 0.043	H-atom parameters constrained
*wR*(*F*^2^) = 0.107	*w* = 1/[σ^2^(*F*_o_^2^) + (0.0597*P*)^2^] where *P* = (*F*_o_^2^ + 2*F*_c_^2^)/3
*S* = 0.99	(Δ/σ)_max_ < 0.001
2336 reflections	Δρ_max_ = 0.58 e Å^−^^3^
217 parameters	Δρ_min_ = −0.35 e Å^−^^3^
Primary atom site location: structure-invariant direct methods	Extinction correction: none

### Special details

**Table:**

Geometry. All e.s.d.'s (except the e.s.d. in the dihedral angle between two l.s. planes) are estimated using the full covariance matrix. The cell e.s.d.'s are taken into account individually in the estimation of e.s.d.'s in distances, angles and torsion angles; correlations between e.s.d.'s in cell parameters are only used when they are defined by crystal symmetry. An approximate (isotropic) treatment of cell e.s.d.'s is used for estimating e.s.d.'s involving l.s. planes.
Refinement. Refinement of *F*^2^ against ALL reflections. The weighted *R*-factor *wR* and goodness of fit *S* are based on *F*^2^, conventional *R*-factors *R* are based on *F*, with *F* set to zero for negative *F*^2^. The threshold expression of *F*^2^ > σ(*F*^2^) is used only for calculating *R*-factors(gt) *etc*. and is not relevant to the choice of reflections for refinement. *R*-factors based on *F*^2^ are statistically about twice as large as those based on *F*, and *R*- factors based on ALL data will be even larger.

### Fractional atomic coordinates and isotropic or equivalent isotropic displacement parameters (Å^2^)

**Table:**

	*x*	*y*	*z*	*U*_iso_*/*U*_eq_	
Mn1	0.26486 (7)	0.43326 (2)	0.53505 (5)	0.01340 (18)	
N1	0.4147 (4)	0.35226 (13)	0.4456 (3)	0.0176 (6)	
N2	0.1642 (4)	0.33159 (13)	0.5808 (3)	0.0166 (6)	
C1	0.5418 (5)	0.36248 (18)	0.3823 (4)	0.0230 (8)	
H1A	0.5722	0.4067	0.3677	0.028*	
C11	0.2366 (5)	0.27625 (16)	0.5373 (4)	0.0182 (7)	
C2	0.6321 (5)	0.31037 (19)	0.3366 (4)	0.0282 (9)	
H2A	0.7216	0.3200	0.2939	0.034*	
C10	0.0358 (5)	0.32165 (18)	0.6425 (4)	0.0223 (8)	
H10A	−0.0171	0.3591	0.6709	0.027*	
C12	0.3729 (5)	0.28764 (16)	0.4675 (4)	0.0196 (8)	
C7	0.1838 (5)	0.21006 (17)	0.5571 (4)	0.0255 (8)	
C4	0.4550 (5)	0.23179 (18)	0.4230 (4)	0.0243 (8)	
C3	0.5875 (5)	0.2453 (2)	0.3552 (4)	0.0285 (9)	
H3A	0.6443	0.2100	0.3233	0.034*	
C5	0.3984 (6)	0.16497 (18)	0.4459 (4)	0.0321 (10)	
H5A	0.4519	0.1280	0.4165	0.039*	
C9	−0.0232 (5)	0.2580 (2)	0.6666 (4)	0.0302 (9)	
H9A	−0.1137	0.2535	0.7105	0.036*	
C6	0.2683 (6)	0.15507 (18)	0.5097 (4)	0.0311 (10)	
H6A	0.2333	0.1112	0.5230	0.037*	
C8	0.0499 (6)	0.20273 (19)	0.6268 (4)	0.0336 (10)	
H8A	0.0125	0.1600	0.6451	0.040*	
C13	−0.0288 (4)	0.49094 (14)	0.1344 (3)	0.0097 (6)	
C16	0.8525 (4)	0.48483 (15)	0.8620 (3)	0.0127 (7)	
C14	−0.0734 (4)	0.48127 (16)	0.2744 (3)	0.0139 (7)	
C17	0.6894 (4)	0.46887 (15)	0.7169 (3)	0.0138 (7)	
C15	0.8273 (4)	0.47604 (15)	0.9971 (3)	0.0132 (7)	
H15A	0.7104	0.4597	0.9953	0.020*	
O1	−0.0214 (3)	0.42752 (11)	0.3466 (2)	0.0190 (5)	
O4	0.5436 (3)	0.44168 (12)	0.7236 (2)	0.0225 (6)	
O3	0.2918 (3)	0.51596 (11)	0.4038 (2)	0.0190 (5)	
O2	0.1650 (3)	0.47176 (12)	0.6976 (2)	0.0235 (6)	

### Atomic displacement parameters (Å^2^)

**Table:**

	*U*^11^	*U*^22^	*U*^33^	*U*^12^	*U*^13^	*U*^23^
Mn1	0.0180 (3)	0.0144 (3)	0.0099 (3)	−0.0002 (2)	0.0077 (2)	0.0001 (2)
N1	0.0191 (16)	0.0201 (16)	0.0154 (15)	0.0002 (12)	0.0083 (12)	−0.0011 (11)
N2	0.0205 (16)	0.0167 (15)	0.0139 (14)	0.0011 (12)	0.0079 (12)	0.0031 (11)
C1	0.023 (2)	0.029 (2)	0.0188 (19)	−0.0053 (16)	0.0099 (16)	−0.0061 (15)
C11	0.024 (2)	0.0169 (17)	0.0111 (17)	−0.0008 (14)	0.0040 (14)	0.0005 (13)
C2	0.026 (2)	0.036 (2)	0.028 (2)	0.0041 (17)	0.0160 (18)	−0.0040 (17)
C10	0.022 (2)	0.027 (2)	0.0185 (19)	−0.0010 (15)	0.0081 (16)	0.0020 (15)
C12	0.0224 (19)	0.0213 (19)	0.0112 (17)	0.0019 (14)	0.0018 (15)	−0.0004 (13)
C7	0.032 (2)	0.0211 (19)	0.0173 (19)	−0.0048 (16)	0.0028 (16)	0.0017 (14)
C4	0.027 (2)	0.0226 (19)	0.0153 (19)	0.0059 (15)	−0.0016 (15)	−0.0059 (14)
C3	0.028 (2)	0.037 (2)	0.0180 (19)	0.0140 (17)	0.0059 (16)	−0.0062 (17)
C5	0.046 (3)	0.019 (2)	0.023 (2)	0.0117 (18)	0.0043 (19)	−0.0017 (15)
C9	0.031 (2)	0.037 (2)	0.024 (2)	−0.0093 (18)	0.0124 (18)	0.0045 (17)
C6	0.052 (3)	0.0132 (18)	0.021 (2)	−0.0037 (17)	0.0054 (19)	0.0024 (14)
C8	0.043 (3)	0.024 (2)	0.028 (2)	−0.0164 (18)	0.0059 (19)	0.0072 (17)
C13	0.0145 (17)	0.0092 (15)	0.0080 (16)	0.0022 (12)	0.0072 (13)	0.0007 (11)
C16	0.0195 (18)	0.0104 (16)	0.0087 (16)	0.0019 (13)	0.0059 (13)	−0.0009 (12)
C14	0.0147 (17)	0.0180 (18)	0.0083 (16)	−0.0016 (14)	0.0037 (13)	−0.0028 (13)
C17	0.0152 (18)	0.0133 (16)	0.0133 (17)	0.0008 (14)	0.0060 (14)	−0.0013 (13)
C15	0.0138 (17)	0.0109 (16)	0.0169 (18)	−0.0037 (13)	0.0079 (14)	−0.0009 (13)
O1	0.0222 (13)	0.0204 (13)	0.0156 (12)	−0.0013 (10)	0.0086 (10)	0.0051 (10)
O4	0.0168 (13)	0.0371 (15)	0.0129 (12)	−0.0094 (11)	0.0046 (10)	−0.0013 (10)
O3	0.0248 (14)	0.0225 (13)	0.0097 (12)	−0.0064 (10)	0.0065 (10)	0.0032 (9)
O2	0.0300 (15)	0.0297 (14)	0.0166 (13)	0.0079 (11)	0.0154 (11)	−0.0008 (10)

### Geometric parameters (Å, °)

**Table:**

Mn1—O2	2.116 (2)	C4—C5	1.433 (5)
Mn1—O3	2.125 (2)	C3—H3A	0.9300
Mn1—O4	2.204 (2)	C5—C6	1.350 (5)
Mn1—O1	2.237 (2)	C5—H5A	0.9300
Mn1—N2	2.252 (3)	C9—C8	1.344 (5)
Mn1—N1	2.305 (3)	C9—H9A	0.9300
N1—C1	1.327 (4)	C6—H6A	0.9300
N1—C12	1.353 (4)	C8—H8A	0.9300
N2—C10	1.323 (4)	C13—C15^i^	1.390 (4)
N2—C11	1.358 (4)	C13—C16^ii^	1.397 (4)
C1—C2	1.394 (5)	C13—C14	1.518 (4)
C1—H1A	0.9300	C16—C15	1.394 (4)
C11—C7	1.404 (5)	C16—C13^ii^	1.397 (4)
C11—C12	1.438 (5)	C16—C17	1.506 (4)
C2—C3	1.360 (5)	C14—O2^iii^	1.246 (4)
C2—H2A	0.9300	C14—O1	1.251 (4)
C10—C9	1.385 (5)	C17—O4	1.244 (4)
C10—H10A	0.9300	C17—O3^ii^	1.259 (4)
C12—C4	1.409 (5)	C15—C13^iv^	1.390 (4)
C7—C8	1.410 (5)	C15—H15A	0.9300
C7—C6	1.420 (5)	O3—C17^ii^	1.259 (4)
C4—C3	1.406 (5)	O2—C14^iii^	1.246 (4)
			
O2—Mn1—O3	107.56 (9)	C8—C7—C6	124.0 (3)
O2—Mn1—O4	81.59 (9)	C3—C4—C12	117.3 (3)
O3—Mn1—O4	99.15 (8)	C3—C4—C5	123.4 (3)
O2—Mn1—O1	96.86 (9)	C12—C4—C5	119.3 (3)
O3—Mn1—O1	80.40 (8)	C2—C3—C4	119.6 (3)
O4—Mn1—O1	178.19 (9)	C2—C3—H3A	120.2
O2—Mn1—N2	86.52 (9)	C4—C3—H3A	120.2
O3—Mn1—N2	156.89 (9)	C6—C5—C4	120.8 (3)
O4—Mn1—N2	101.02 (9)	C6—C5—H5A	119.6
O1—Mn1—N2	79.78 (9)	C4—C5—H5A	119.6
O2—Mn1—N1	152.37 (9)	C8—C9—C10	120.2 (3)
O3—Mn1—N1	98.36 (9)	C8—C9—H9A	119.9
O4—Mn1—N1	85.10 (9)	C10—C9—H9A	119.9
O1—Mn1—N1	96.70 (9)	C5—C6—C7	121.5 (3)
N2—Mn1—N1	72.38 (9)	C5—C6—H6A	119.2
C1—N1—C12	117.7 (3)	C7—C6—H6A	119.2
C1—N1—Mn1	127.0 (2)	C9—C8—C7	119.5 (3)
C12—N1—Mn1	115.2 (2)	C9—C8—H8A	120.2
C10—N2—C11	117.5 (3)	C7—C8—H8A	120.2
C10—N2—Mn1	125.1 (2)	C15^i^—C13—C16^ii^	119.2 (3)
C11—N2—Mn1	117.3 (2)	C15^i^—C13—C14	117.8 (3)
N1—C1—C2	123.5 (3)	C16^ii^—C13—C14	123.0 (3)
N1—C1—H1A	118.3	C15—C16—C13^ii^	118.6 (3)
C2—C1—H1A	118.3	C15—C16—C17	119.5 (3)
N2—C11—C7	123.1 (3)	C13^ii^—C16—C17	121.9 (3)
N2—C11—C12	117.1 (3)	O2^iii^—C14—O1	126.7 (3)
C7—C11—C12	119.8 (3)	O2^iii^—C14—C13	115.0 (3)
C3—C2—C1	119.1 (3)	O1—C14—C13	118.3 (3)
C3—C2—H2A	120.4	O4—C17—O3^ii^	123.8 (3)
C1—C2—H2A	120.4	O4—C17—C16	118.0 (3)
N2—C10—C9	123.0 (3)	O3^ii^—C17—C16	118.2 (3)
N2—C10—H10A	118.5	C13^iv^—C15—C16	122.2 (3)
C9—C10—H10A	118.5	C13^iv^—C15—H15A	118.9
N1—C12—C4	122.8 (3)	C16—C15—H15A	118.9
N1—C12—C11	118.0 (3)	C14—O1—Mn1	114.4 (2)
C4—C12—C11	119.2 (3)	C17—O4—Mn1	125.0 (2)
C11—C7—C8	116.7 (3)	C17^ii^—O3—Mn1	143.3 (2)
C11—C7—C6	119.3 (3)	C14^iii^—O2—Mn1	143.9 (2)
			
O2—Mn1—N1—C1	−136.3 (3)	C1—C2—C3—C4	−1.4 (5)
O3—Mn1—N1—C1	23.6 (3)	C12—C4—C3—C2	0.6 (5)
O4—Mn1—N1—C1	−75.0 (3)	C5—C4—C3—C2	179.4 (3)
O1—Mn1—N1—C1	104.8 (3)	C3—C4—C5—C6	−179.0 (3)
N2—Mn1—N1—C1	−178.2 (3)	C12—C4—C5—C6	−0.2 (5)
O2—Mn1—N1—C12	40.5 (3)	N2—C10—C9—C8	−0.1 (5)
O3—Mn1—N1—C12	−159.7 (2)	C4—C5—C6—C7	−0.3 (6)
O4—Mn1—N1—C12	101.7 (2)	C11—C7—C6—C5	−0.1 (6)
O1—Mn1—N1—C12	−78.5 (2)	C8—C7—C6—C5	−178.8 (3)
N2—Mn1—N1—C12	−1.5 (2)	C10—C9—C8—C7	−1.5 (5)
O2—Mn1—N2—C10	20.7 (3)	C11—C7—C8—C9	1.7 (5)
O3—Mn1—N2—C10	−108.2 (3)	C6—C7—C8—C9	−179.5 (3)
O4—Mn1—N2—C10	101.4 (3)	C15^i^—C13—C14—O2^iii^	−80.8 (4)
O1—Mn1—N2—C10	−77.0 (3)	C16^ii^—C13—C14—O2^iii^	97.9 (4)
N1—Mn1—N2—C10	−177.4 (3)	C15^i^—C13—C14—O1	97.1 (4)
O2—Mn1—N2—C11	−161.6 (2)	C16^ii^—C13—C14—O1	−84.2 (4)
O3—Mn1—N2—C11	69.5 (3)	C15—C16—C17—O4	−7.3 (4)
O4—Mn1—N2—C11	−80.8 (2)	C13^ii^—C16—C17—O4	173.5 (3)
O1—Mn1—N2—C11	100.8 (2)	C15—C16—C17—O3^ii^	172.5 (3)
N1—Mn1—N2—C11	0.3 (2)	C13^ii^—C16—C17—O3^ii^	−6.8 (4)
C12—N1—C1—C2	0.6 (5)	C13^ii^—C16—C15—C13^iv^	0.4 (5)
Mn1—N1—C1—C2	177.2 (3)	C17—C16—C15—C13^iv^	−178.9 (3)
C10—N2—C11—C7	−1.1 (5)	O2^iii^—C14—O1—Mn1	−88.5 (4)
Mn1—N2—C11—C7	−179.0 (3)	C13—C14—O1—Mn1	93.8 (3)
C10—N2—C11—C12	178.7 (3)	O2—Mn1—O1—C14	85.7 (2)
Mn1—N2—C11—C12	0.8 (4)	O3—Mn1—O1—C14	−21.1 (2)
N1—C1—C2—C3	0.9 (5)	N2—Mn1—O1—C14	170.9 (2)
C11—N2—C10—C9	1.4 (5)	N1—Mn1—O1—C14	−118.5 (2)
Mn1—N2—C10—C9	179.1 (2)	O3^ii^—C17—O4—Mn1	−13.7 (4)
C1—N1—C12—C4	−1.5 (5)	C16—C17—O4—Mn1	165.99 (19)
Mn1—N1—C12—C4	−178.6 (3)	O2—Mn1—O4—C17	−130.8 (3)
C1—N1—C12—C11	179.4 (3)	O3—Mn1—O4—C17	−24.2 (3)
Mn1—N1—C12—C11	2.4 (4)	N2—Mn1—O4—C17	144.5 (3)
N2—C11—C12—N1	−2.1 (5)	N1—Mn1—O4—C17	73.5 (3)
C7—C11—C12—N1	177.6 (3)	O2—Mn1—O3—C17^ii^	−164.7 (3)
N2—C11—C12—C4	178.8 (3)	O4—Mn1—O3—C17^ii^	111.3 (3)
C7—C11—C12—C4	−1.4 (5)	O1—Mn1—O3—C17^ii^	−70.5 (3)
N2—C11—C7—C8	−0.4 (5)	N2—Mn1—O3—C17^ii^	−39.2 (5)
C12—C11—C7—C8	179.8 (3)	N1—Mn1—O3—C17^ii^	25.0 (4)
N2—C11—C7—C6	−179.3 (3)	O3—Mn1—O2—C14^iii^	25.6 (4)
C12—C11—C7—C6	0.9 (5)	O4—Mn1—O2—C14^iii^	122.6 (4)
N1—C12—C4—C3	1.0 (5)	O1—Mn1—O2—C14^iii^	−56.4 (4)
C11—C12—C4—C3	180.0 (3)	N2—Mn1—O2—C14^iii^	−135.7 (4)
N1—C12—C4—C5	−178.0 (3)	N1—Mn1—O2—C14^iii^	−175.3 (3)
C11—C12—C4—C5	1.1 (5)		

Symmetry codes: (i) *x*−1, *y*, *z*−1; (ii) −*x*+1, −*y*+1, −*z*+1; (iii) −*x*, −*y*+1, −*z*+1; (iv) *x*+1, *y*, *z*+1.
